# A morphological classification for vocal fold leukoplakia^☆^

**DOI:** 10.1016/j.bjorl.2018.04.014

**Published:** 2018-06-11

**Authors:** Min Chen, Changjiang Li, Yue Yang, Lei Cheng, Haitao Wu

**Affiliations:** a Fudan University, Eye, Ear, Nose, and Throat Hospital, Department of Otolaryngology-Head and Neck Surgery, Shanghai, China; b Shanghai Key Clinical, Disciplines of Otorhinolaryngology, Shanghai, China

**Keywords:** Vocal fold, Leukoplakia, Morphological, Pathological, Dysplasia, Prega vocal, Leucoplasia, Morfológico, Patológico, Displasia

## Abstract

**Introduction:**

There is still no general method for discriminating between benign and malignant leukoplakia and identifying vocal fold leukoplakia.

**Objective:**

To evaluate the reliability of a morphological classification and the correlation between morphological types and pathological grades of vocal fold leukoplakia.

**Methods:**

A total of 375 patients with vocal fold leukoplakia between 2009 and 2015 were retrospectively reviewed. Two observers divided the vocal fold leukoplakia into flat and smooth, elevated and smooth, and rough type on the basis of morphological appearance. The inter-observer reliability was evaluated and the results of classification from both observers were compared with final pathological grades. Clinical characteristics between low risk and high risk group were also analyzed.

**Results:**

The percentage inter-observer agreement of the morphological classification was 78.7% (*κ* = 0.615, *p* < 0.001). In the results from both observers, the morphological types were significantly correlated with the pathological grades (*p*_1_ < 0.001, *p*_2_ < 0.001, Kruskal–Wallis test; *r*_1_ = 0.646, *p*_1_ < 0.001, *r*_2_ = 0.539, *p*_2_ < 0.001, Spearman Correlation Analysis). Multivariate analysis showed patient's age (*p* = 0.018), the size of lesion (*p* < 0.001), and morphological type (*p* < 0.001) were significantly different between low risk group and high risk group. Combined receiver operating characteristic curve analysis of significant parameters revealed an area under the receiver operating characteristic curve of 0.863 (95% CI 0.823–0.903, *p* < 0.001).

**Conclusions:**

The proposed morphological classification of vocal fold leukoplakia was consistent between observers and morphological types correlated with pathological grades. Patient's age, the size of lesion, and morphological type might enable risk stratification and provide treatment guidelines for vocal fold leukoplakia.

## Resumo

**Introdução:**

Ainda não há um método universal estabelecido para diferenciar entre a leucoplasia benigna e maligna ou identificar as leucoplasias das pregas vocais.

**Objetivo:**

Avaliar a confiabilidade de uma classificação morfológica e a correlação entre os tipos morfológicos e os graus histopatológicos das leucoplasias de pregas vocais.

**Método:**

Os registros de 375 pacientes com leucoplasia da prega vocal assistidos entre 2009 e 2015 foram revisados retrospectivamente. Dois observadores dividiram a leucoplasia da prega vocal entre tipo plano e liso, elevado e liso, e rugoso, com base na aparência morfológica. A confiabilidade interobservador foi avaliada e os resultados de classificação de ambos os observadores foram comparados com os graus histopatológicos finais. As características clínicas entre os grupos de baixo risco e alto risco também foram analisadas.

**Resultados:**

A porcentagem da concordância inter-observador da classificação morfológica foi de 78,7% (κ = 0,615, *p* < 0,001). Nos resultados de ambos os observadores, os tipos morfológicos correlacionaram-se significativamente com os graus histopatológicos (*p*_1_ < 0,001, *p*_2_ < 0,001, teste de Kruskal-Wallis; r_1_ = 0,646, *p*_1_ < 0,001, r_2_ = 0,539, *p*_2_ < 0,001, análise de correlação de Spearman). A análise multivariada mostrou que a idade do paciente (*p* = 0,018), o tamanho da lesão (*p* < 0,001) e o tipo morfológico (*p* < 0,001) foram significativamente diferentes entre o grupo de baixo risco e o de alto risco. A análise da curva ROC (*Receiver Operating Characteristic*) combinada de parâmetros significativos revelou uma área sob a curva de 0,863 (IC 95%: 0,823 ± 0,903, *p* < 0,001).

**Conclusões:**

A classificação morfológica proposta para leucoplasia de prega vocal foi consistente entre observadores e os tipos morfológicos correlacionaram-se com os graus histopatológicos. A idade do paciente, o tamanho da lesão e o tipo morfológico podem permitir a estratificação de risco e fornecem diretrizes de tratamento para a leucoplasia da prega vocal.

## Introduction

Vocal fold leukoplakia is an abnormal mucosa lesion with flat or thick epithelial white plaques or patches that cannot be defined as any other condition.^1^ Pathological changes remain the mainstays of accurate diagnosis and decisive guidance for management of vocal fold leukoplakia.^2^ The dysplasia system (DS), a 5 grade pathological classification for vocal fold leukoplakia, including non-dysplasia, mild dysplasia, moderate dysplasia, severe dysplasia, and carcinoma, is the most frequently used.^3^, ^4^ With a trend in other organs to a 2 grade system, a two-tier classification, such as “no/mild dysplasia” – low risk, and “moderate or severe” – high risk, was introduced in the WHO 2017 Blue Book.^5^

There is still no consensus on the treatment of vocal fold leukoplakia.^1^ The clinical importance for vocal fold leukoplakia lies in its tendency to transform into invasive carcinoma. Reported malignant transformation rates of laryngeal dysplasia vary between 11% and 25%, but identifying which patients would transform into carcinoma is complicated.^6^, ^7^ For those leukoplakia patients without dysplasia, excisional treatment is not required. And immediate surgical treatments are not considered for patients with mild dysplastic vocal fold leukoplakia because of their low risk for malignant transformation in the short term.^8^ However, there is no objective nonsurgical method available for evaluating the degree of dysplasia and the presence of malignant change; clinicians often are concerned that not doing enough may lead to progression to invasive laryngeal squamous cell carcinoma (LSCC). Surgical therapy still remains the most widely studied modality of treatment.^9^, ^10^ Postoperative pathological analysis revealed distinct rates of dysplasia reported by different groups. Isenberg et al.^11^ and Cui et al.^12^ reported that approximately 50% of patients clinically diagnosed with vocal fold leukoplakia do not have dysplasia, indicating that these patients received unnecessary surgical treatment. For this reason, we search for a non-invasive tool for discriminating benign and malignant leukoplakia and identifying appropriate non-surgical or surgical for leukoplakia.

Narrow band imaging (NBI) endoscopy is one of the modern tools that improve the evaluation of laryngeal lesions, which is employed for distinguishing between benign and malignant patterns of vocal cord leukoplakia.^13^, ^14^ However, NBI does not focus on the biological features of the leukoplakia itself, but highlights its vascularization.^15^ Visual obstruction by a thick keratin layer covering on the vocal cord also limits its application.^16^ Nevertheless, normal laryngoscopy under white Llght (WL) was the most common tool for vocal fold leukoplakia, which displays the lesion more directly and clearly.

Currently, the studies about the classification of vocal fold leukoplakia by morphological characteristics are very limited,^8^, ^17^ which jeopardizes establishment of a standard treatment for vocal fold leukoplakia. A reliable and practical approach is required to better classify the vocal fold leukoplakia by morphological characteristics and identify the degree of dysplasia.

Here we propose a new classification for vocal fold leukoplakia based on the morphological changes under laryngoscopy. The aim of this study was to evaluate the reliability of this morphological classification and the correlation between the morphological types and pathological grades of vocal fold leukoplakia. We attempted to address whether the morphological types could reflect the pathological grades. To fulfill our goal, a clinicopathological study of 375 cases with vocal fold leukoplakia was conducted.

## Methods

### Patients

The protocol of this retrospective study was approved by the Institution Review Board of the Eye, Ear, Nose and Throat Hospital of Fudan University, Shanghai, China (Approval n° 2017042-1). Patients for this study were collected from February 2009 to June 2015. A total of 604 outpatients were primarily diagnosed as vocal fold leukoplakia according to office-based rigid laryngoscopy. Of them, 375 patients included in this study underwent microlaryngeal surgery and were pathologically diagnosed with keratosis or dyskeratosis with hyperplasia, non-dysplasia, dysplasia or carcinoma. Patients without preoperative images of office-based laryngoscope examination, surgical removal of lesions, or postoperative pathological records were excluded.

### Clinical data

Clinical data, including age, gender, preoperative laryngoscope images, and postoperative pathological records, were collected. The size of lesion was recorded as <50% (the sum of all leukoplakia is less than half length of one entire vocal cord) or >50% (the sum of all leukoplakia is more than half length of one entire vocal cord).^8^, ^17^ The involvement of the anterior commissure was defined as leukoplakia was located at the anterior commissure of vocal cords.

### Morphological types

**Figure 1:**
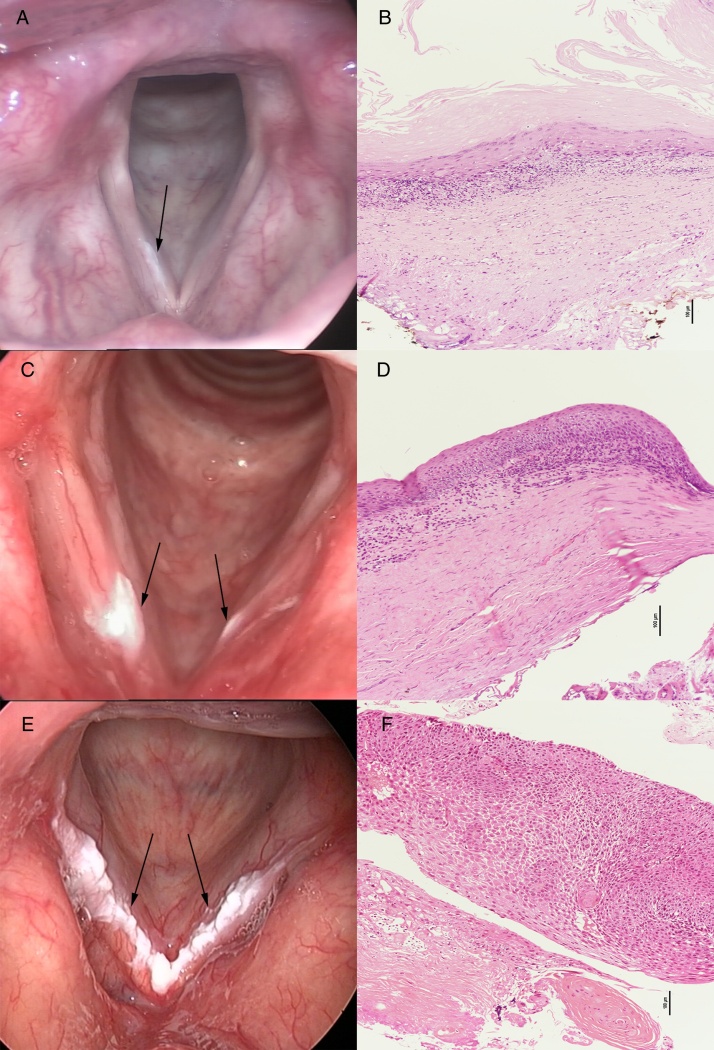
The pathological results of flat and smooth leukoplakia (A), elevated and smooth leukoplakia (C), and rough leukoplakia (E) showed squamous hyperplasia without dysplasia (B), squamous hyperplasia with mild-dysplasia (D), and squamous cell carcinoma (F), respectively.

The morphological types of vocal fold leukoplakia assessed by preoperative rigid laryngoscopy were categorized as: flat and smooth, elevated and smooth, and rough type.^18^ The definition is presented as the following:

Flat and smooth type: Surface: smooth; Margin: lesion without raised margins, being continuous with the surrounding mucosa; Texture: homogeneous, regular, the lesion with even coloration (Fig. 1A).

Elevated and smooth type: Surface: smooth; Margin: lesion with raised margins, sharply demarcated from the surrounding mucosa; Texture: homogeneous, regular, the lesion with even coloration (Fig. 1C).

Rough type: Surface: wrinkled, corrugated; Margin: lesion with raised margins, sharply demarcated from the surrounding mucosa; Texture: non-homogeneous, irregular, the lesion with uneven coloration and is usually accompanied with erosion or ulceration (Fig. 1E).

When various types of leukoplakia coexist, elevated and smooth leukoplakia will be determined if flat and smooth and elevated and smooth leukoplakia on vocal cords; rough leukoplakia will be determined once rough lesion appears on vocal cords.

Two observers, an experienced laryngologist and an otolaryngology resident, blinded to the patients’ clinical information and postoperative pathological results, independently classified each preoperative laryngoscope image according to the definition mentioned above. The inter-observer reliability of the morphological classification was evaluated.

### Treatment

Written informed consent for surgery was signed by each patient. Endotracheal intubation and the full glottic lesions were visualized with a binocular microscope (ZEISS S88, Carl Zeiss Shanghai Co.). Complete resection by CO_2_ laser (Lumenis 40C, Yokneam 20692, Israel) was performed for all patients under general anesthesia. Each patient was discharged from hospital the next day after surgery. Patients who had bilateral leukoplakia were treated with staged procedures. Once the postoperative pathological result showed squamous cell carcinoma, further radical surgery would be arranged. Follow-up appointments of postoperative management were arranged every 1–3 months for the first year and then every 3–6 months for the following years. Patients were assessed by office-based rigid laryngoscope (Panasonic GP-KS822) examination.

### Histological assessment

All the tissues were routinely processed for pathological examination. Formalin-fixed and paraffin-embedded slides were independently viewed and histologically graded by three pathologists in the Department of Pathology at Eye, Ear, Nose and Throat Hospital of Fudan University, Shanghai, China. Histological assessment was conducted according to the World Health Organization 2005 guidelines^3^ in which vocal fold leukoplakia is divided into the following categories: squamous cell hyperplasia with non-dysplasia, mild dysplasia, moderate dysplasia, severe dysplasia, carcinoma in situ and squamous cell carcinoma. Squamous cell hyperplasia with non-dysplasia describes increased cell numbers but the architecture shows regular stratification and there is no cellular atypia. Mild dysplasia describes slight cytological atypia, most marked in the basal one-third of the epithelium. Moderate dysplasia describes more cytological atypia, changes presenting in the lower two-thirds of the epithelium. Severe dysplasia describes cytological atypia involving more than two-thirds of the epithelial thickness. Carcinoma describes full thickness architectural abnormalities in the viable cellular layers accompanied with cytologic atypia.^3^, ^4^

### Statistical analysis

Statistical analysis of the data was performed using SPSS software version 23.0 (IBM Corporation, 2015, USA). The inter-observer reliability of the morphological classification was determined using the Kappa test. The Kruskal–Wallis test and Spearmen Correlation Analysis were used to analyze pathological grades of flat and smooth, elevated and smooth, and rough leukoplakia identify by both observers, respectively. The proportion of non-dysplasia and carcinoma in three types of leukoplakia was calculated, respectively. The morphological classification and other clinical parameters were compared between low risk group and high risk group by univariate analysis and multivariate logistic analysis. Clinical parameters were analyzed by Student's *t* test for continuous variables, Chi-square test for binary variables, and Kruskal–Wallis test followed by Nemenyi test for those consisted of several independent groups. The diagnostic accuracy of clinical characteristics of vocal fold leukoplakia was evaluated using the area under the receiver operating characteristics (ROC) curve (AUC) analysis and logistic regression. Two-sided *p*-values < 0.05 were considered significant.

## Results

A total of 375 patients diagnosed with vocal fold leukoplakia were included in this study, of whom 364 (97.1%) were male and 11 (2.9%) were female. The average age of the patients was 57.6 ± 9.6 years with an age range of 31–86 years. Characteristics of baseline patient information were listed in Table 1. The results of 5 grades and 2 grade pathological classifications were listed in Table 2.

**Table 1:** Clinical characteristics of patients with vocal fold leukoplakia.

Variables	Total	Low risk	High risk	*p*
*Age (years)*		53.46 ± 10.05	59.34 ± 8.89	<0.001^a^
<60	218 (58.4%)	83	135	<0.001^b^
≥60	157 (41.6%)	28	129	

*Gender*				0.400^b^
Male	364 (97.1%)	109	255	
Female	11 (2.9%)	2	9	

*Site*				0.395^b^
Unilateral	283 (75.5%)	87	196	
Bilateral	92 (24.5%)	24	68	

*Size*				<0.001^b^
<50%	252 (67.2%)	107	15	
≥50%	123 (32.8%)	4	119	

*Anterior commissure involved*				0.126^b^
Yes	16 (4.3%)	2	14	
No	359 (95.7%)	109	250	

*Clinical type*				
Flat and smooth	25 (6.7%)	25	0	*p*_1_ < 0.001^c^
Elevated and smooth	154 (41.0%)	71	83	*p*_2_ < 0.001^c^
Rough	196 (52.3%)	15	181	*p*_3_ < 0.001^c^

a Student's *t* test.

b Chi-square test.

c Kruskal–Wallis test followed by Nemenyi test (*p*_1_, flat and smooth vs. elevated and smooth; *p*_2_, elevated and smooth vs. rough; *p*_3_, rough vs. flat and smooth).

**Table 2:** Pathological grade of vocal fold leukoplakia in 375 cases.

Pathological grade	Number of patients (%)
Low risk group	111 (29.6%)
Non-dysplasia	39 (10.4%)
Mild-dysplasia	72 (19.2%)
High risk group	264 (70.4%)
Moderate-dysplasia	51 (13.6%)
Severe-dysplasia	145 (38.7%)
Squamous cell carcinoma	68 (18.1%)

375 preoperative laryngoscope images were identified by two observers (Table 3). The percentage inter-observer agreement between the experienced laryngologist and the otolaryngology resident was 78.7% (*κ*-value = 0.615, *p* < 0.001).

**Table 3:** Morphological and pathological diagnosis of vocal fold leukoplakia.

	Flat and smooth		Elevated and smooth		Rough		p	
	Observer 1	Observer 2	Observer 1	Observer 2	Observer 1	Observer 2	Observer 1	Observer 2
Non-dysplasia	17	13	20	22	2	4		
Mild-dysplasia	8	10	51	46	13	16	<0.001^a^	<0.001^a^
Moderate-dysplasia	0	3	39	32	12	16	<0.001^b^	<0.001^b^
Severe-dysplasia	0	2	36	35	109	108	<0.001^c^	<0.001^c^
Carcinoma	0	0	8	10	60	58	<0.001^d^	<0.001^d^
Total	25	28	154	145	196	202		

a Kruskal–Wallis test followed by Nemenyi test, flat and smooth vs. elevated and smooth.

b Kruskal–Wallis test followed by Nemenyi test, flat and smooth vs. rough.

c Kruskal–Wallis test followed by Nemenyi test, elevated and smooth vs. rough.

d Spearman Correlation Analysis.

In the results from both observers (Table 3), significant differences in pathological grades were witnessed among three morphological types according to Kruskal–Wallis test followed by the Nemenyi test (flat and smooth vs. elevated and smooth, *p*_1_ < 0.001 and *p*_2_ < 0.001; flat and smooth vs. rough, *p*_1_ < 0.001 and *p*_2_ < 0.001; elevated and smooth vs. rough, *p*_1_ < 0.001 and *p*_2_ < 0.001, respectively). Morphological types revealed a significant correlation with pathological grades by Spearman Correlation Analysis (*r*_1_ = 0.553, *p*_1_ < 0.001; *r*_2_ = 0.498, *p*_2_ < 0.001, respectively) (*r*_1_, *p*_1_, observer 1; *r*_2_, *p*_2_, observer 2).

Based on the consistent results between two observers, the following data was evaluated by the experienced laryngologist (Observer 1). The incidence of non-dysplasia in flat and smooth, elevated and smooth, and rough leukoplakia was 68%, 13.0%, 1.0%; the incidence of carcinoma in flat and smooth, elevated and smooth, and rough leukoplakia was 0%, 5.2%, 30.6%.

**Figure 2:**
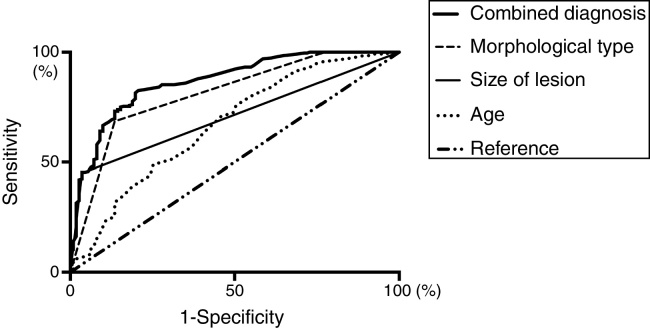
Receiver Operating Characteristic (ROC) analysis and the Areas Under the Curve (AUCs) of independent predictors for distinguishing low risk and high risk of vocal fold leukoplakia.

Clinical characteristics were compared between low risk group and high risk group univariate analysis was listed in Table 1. Age (Odd Ratio = 0.495, 95% CI 0.276–0.885, *p* = 0.018), the size of lesion (Odd Ratio = 0.102, 95% CI = 0.034–0.300, *p* < 0.001), and morphological type (Odd Ratio = 0.145, 95% CI 0.076–0.278, *p* < 0.001) were significantly different between two groups testified by multivariate logistic regression analysis. The AUC for age, size of lesion, and morphological type were 0.670 (95% CI 0.608–0.732, *p* < 0.001), 0.707 (95% CI 0.655–0.759, *p* < 0.001), and 0.811 (95% CI 0.762–0.859, *p* < 0.001), respectively. Combined receiver operating characteristic curve analysis of significant parameters revealed an AUC of 0.863 (95% CI 0.823–0.903, *p* < 0.001) (Fig. 2).

## Discussion

Vocal fold leukoplakia encompasses a variety of benignities, premalignancies, and malignancies.^19^ Although new endoscopic tools, narrow band imaging, optical coherence tomography and contact endoscope have been developed to improve the distinction for vocal fold leukoplakia, the WL laryngoscopy is more commonly applied in clinical practice.^13^, ^20^, ^21^ The ability of rigid or flexible laryngoscopy to visualize and characterize lesions of vocal cords continues to improve. Our current office laryngoscopes allow precise identification of surface, margin, texture, and size of lesion. In some reports, vocal fold leukoplakia was divided into three morphological groups: superficial type, exophytic type, and ulcerative type or stratified by morphological characteristics scoring.^8^, ^17^, ^22^ In line with our recent studies,^23^, ^24^ we have proposed a new, simple classification for vocal fold leukoplakia on the basis of morphological characteristics visualized by normal laryngoscopy under white light. In our study, 375 laryngoscope images were classified into three types by an experienced laryngologist and an otolaryngology resident independently, blinded to the patients’ clinical characteristics and postoperative pathological results. There were consistent findings of acceptable inter-observer reliability of morphological classification for vocal fold leukoplakia (Table 3). It was found that there was a moderate agreement between two observers. This finding might lead us to assume that this morphological classification proposing a uniform description of vocal fold leukoplakia can be utilized in clinical practice. Furthermore, this classification method we proposed was based on macroscopic appearance visualized by normal laryngoscopy, which might be promoted and popularized to various institutions.

Recently macroscopic features of vocal fold leukoplakia have been associated with pathological changes which remain the mainstay of accurate diagnosis, prognosis, and guidance for management.^2^, ^4^ We classified three types as initial, middle, and advanced stage based on the macroscopic features. In the results from both observers, morphological characteristics revealed a significant correlation with pathological grades of vocal fold leukoplakia after Kruskal–Wallis test and Spearman Correlation Analysis. This relationship has also been suggested in another study.^22^ And the same appeared in leukoplakia in other sites. Homogenous and non-homogenous oral leukoplakia have distinct pathological characteristics and the risk of dysplasia increased in patients presenting the non-homogenous oral leukoplakia.^25^, ^26^, ^27^ Moreover, morphology of superficial mucosal microvessels under NBI was considered to relate to the degree of dysplasia for vocal leukoplakia.^28^ Further study would focus on the relationship among morphological characteristics of lesion itself, mucosal microvessels, and pathological characteristics. The combination of WL and NBI laryngoscopy might benefit in accurate diagnosis for vocal fold leukoplakia.

Post-operative pathological analysis revealed distinct rates of dysplasia reported by different groups. Isenberg^11^ found that 53% of 208 hyperkeratotic vocal fold lesions may not contain dysplasia; Cui^12^ reported that 54.2% of 555 cases with vocal fold leukoplakia was hyperplasia without dysplasia; Yang^29^ and Zhu^30^ noted that 54.5% and 61.6%, respectively, of patients with vocal fold leukoplakia was non-dysplasia. Whereas, in the present study, the rate of non dysplasia for vocal fold leukoplakia was only found in 39 (10.4%) of 375 cases, which is significantly lower than the findings of authors mentioned previously. Different management strategies for the three morphological types of vocal fold leukoplakia we applied might contribute to the lower rate of non-dysplasia. In the flat and smooth, elevated and smooth, and rough group, the proportion of non-dysplasia gradually decreased; on the contrary, the proportion of carcinoma gradually increased. Thus, conservative treatment and close observation were recommended for patients with flat and smooth leukoplakia and elevated and smooth leukoplakia; surgery was only considered when conservative treatment turned out to be ineffective. Surgical treatment was performed for patients with rough leukoplakia as soon as possible.

Clinicians are often concerned that not doing enough may result in progression to invasive LSCC, whereas overtreatment in vocal fold leukoplakia that would not progress may cause scars of the vocal cords and voice deterioration. Discrimination between benign and malignant lesions without a histological evaluation is not possible.^31^ To date, surgical therapy remains the most widely studied modality of treatment and excision of lesions is required before histological diagnosis.^9^, ^10^ Therefore, numerous leukoplakia with non-dysplasia that does not convey premalignant potential received unnecessary surgical treatment in clinical practice. One of the reasons was lack of standards to evaluate the degree of dysplasia for vocal fold leukoplakia. In the present study, we proposed the morphological classification aimed to evaluate the pathological grades for vocal fold leukoplakia. As we reported previously, the effectiveness of non-surgical treatment for flat and smooth and elevated and smooth vocal fold leukoplakia is superior in comparison to rough vocal fold leukoplakia. The complete response rate of non-surgical treatment for flat and smooth leukoplakia was up to 80.3%.^18^ Therefore, this classification method might be helpful in guiding management for vocal fold leukoplakia.

To our knowledge, few studies have focused on correlating clinical appearance with pathological changes for vocal fold leukoplakia. Contrary to the findings that the surface appearance of the leukoplakia was not a reliable prognosticator of the severity of dysplasia,^32^ we found the microscopic appearance correlated with pathological grades. Different classification methods might lead to the discrepancy. The appearance of leukoplakia was evaluated based on merely the surface of lesion by Zeitels et al.,^32^ while our classification including three parameters: surface, margin, and texture. In line with the ideas of previous studies,^22^, ^25^, ^26^, ^27^ we believe texture could not be ignored for evaluating leukoplakia. Additionally, the sample sizes of Zeitels et al.’s and our studies were 52 patients versus 375 patients, and differences in the power of the analyses might be another reason to explain the different findings of both studies. Our finding was in accordance with Fang et al.’s results^22^ that patient's age and laryngoscopic characteristics were the independent predictive factor for risk stratification. However, we believed the size of lesion was another important predictive factor. A consensus statement presented by otorhinolaryngologists and pathologists, produced at a meeting on the diagnosis and management of laryngeal dysplasia in 2010, made the recommendation that the overall appearance of the lesion was considered to be the most important factor in determining management.^9^ Several studies have demonstrated size of lesion was a factor predictive of malignant transformation in patients with leukoplakia.^17^, ^33^ The modern concepts of carcinogenesis have emphasized the existence of a molecularly altered precancerous field from which extensive lesions would develop.^34^, ^35^ Vocal fold leukoplakia categorized as low risk group and high risk group in our study differed from the previous classification in the literature.^22^, ^23^, ^24^ The risk stratification was based on the two-tier classification introduced in the WHO 2017 Blue Book,^5^ which was confirmed to have better inter-rater agreement than had been found in previous studies. A high risk group is associated with invasive carcinoma development in up to 40% of cases, whereas low-risk group shows malignant progression in only approximately 2% of cases.^10^, ^36^, ^37^ Thus, such a distinction can facilitate clinical decisions about treatment modalities for patients with laryngeal lesions.

Additionally, we combined the age, the size of lesion, and morphological type together to assess the predictive risk value. And ROC analysis exhibited a high AUC for predicting pathological risk (Fig. 2). It was the first time that combination of age, the size of lesion, and morphological type were observed to possess a better value in predicting risk, which reinforced our previous achievements.^23^, ^24^ Therefore, the morphological type combined with age and size of lesion should be considered into stratifying risk for vocal fold leukoplakia, and a proper therapy strategy would be developed afterward.

The strengths of this study include creation of a new classification for vocal fold leukoplakia, a comparatively larger series to determine whether laryngoscopic types are related to the pathological grades and suggestion for treatment options based on clinical characteristics. There are still several weaknesses in the present study. Firstly, the fundamental flaw is the retrospective nature of this study. A prospective research is needed to confirm the use of the classification method. Secondly, the inter-observer reliability produced a disagreement of 21.3%; inexperienced otolaryngology residents need to be trained before applying this classification into clinical practice. Thirdly, patients with vocal fold leukoplakia included in the study are only those who received surgical treatments. These lesions might be more serious so that couldn’t represent the condition for all the lesions. Besides, we were not able to investigate the predictive factors for pathological grades of lesions by combining other specific factors, including tobacco smoking, alcohol intake and laryngopharyngeal reflux. Whether the morphological type of vocal fold leukoplakia is related to the malignant transformation and recurrence also deserves to be studied.

## Conclusion

Vocal fold leukoplakia could be categorized as flat and smooth, elevated and smooth, and rough type on the basis of the morphological classification, which was shown to be consistent between observers. The morphological types of vocal fold leukoplakia correlated with pathological grades. Patient's age, the size of lesion, and morphological type might enable risk stratification and provide treatment guidelines for vocal fold leukoplakia.

## Funding

The work was supported by the Science and Technology Commission of Shanghai Municipality of China (Grant n° 15401971600 and Grant n° 17411962000) and also supported by the Health and Family Planning Commission of Shanghai Municipality of China (Grant n° 2016LP19). This work did not involve the use of human subjects or animal.

## Conflicts of interest

The authors declare no conflicts of interest.
